# Supplementary material for: Tryptase β regulation of joint lubrication and inflammation via proteoglycan-4 in osteoarthritis
Source: Nat Commun. 2023 Apr 6;14:1910. doi: 10.1038/s41467-023-37598-3 (PMC10079686; doi:10.1038/s41467-023-37598-3)
Supplement: Supplementary file 1 — Supplementary Information [file 41467_2023_37598_MOESM1_ESM.pdf]

a

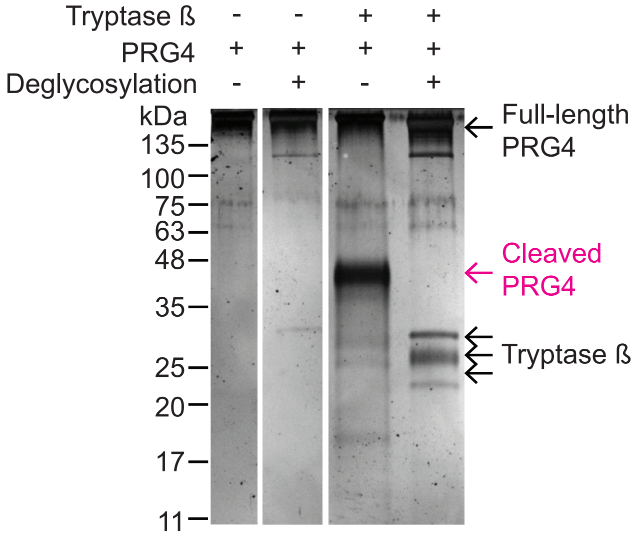

b

ATOMS workflow for1 hour and 24 hours

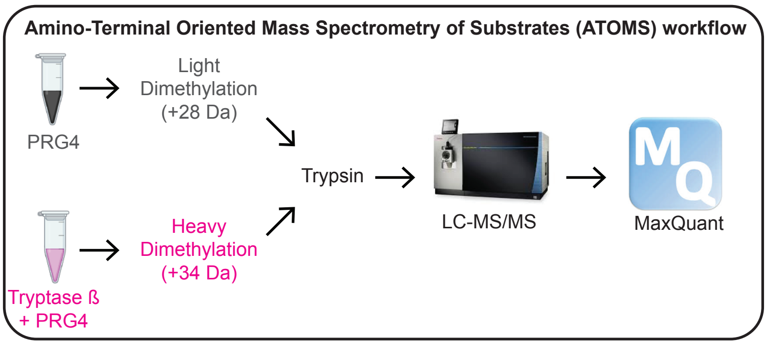

c

Cleavage sites at 1 hour and 24 hours

PRG4 Peptides	cleavage site
1 hour	
RVCTAELSCK	61K↓R62
VCTAELSCK	62R↓V63
ECDCDAQCKKYDK	82R↓E83
KAPPPSGASQTIK	117K↓K118
TKKVIESEEITEEHSVSENQESSSSSSSSSSSTIR	144K↓T145
KVIESEEITEEHSVSENQESSSSSSSSSSSTIR	146K↓K147
VIESEEITEEHSVSENQESSSSSSSSSSSTIR	147K↓V148
ETSLTVNK	272K↓E273
ETTVETKETTTTNK	280K↓E281
ETTTTNK	287K↓E288
TTSAKETQSIEK	303K↓T304
ETQSIEK	308K↓E309
DLAPTSK	319K↓D320
SAPTTPKEPAPTTPK	372K↓S373
GTAPTTLK	710K↓G711
SEDAGGAEGETPHMLLR	1093K↓S1094
CNCEGK	1201R↓C1202
DSQYWR	1212K↓D1213
FTNDIK	1218R↓F1219
DAGYPKPIFK	1224K↓D1225
RGGSIQQYIYK	1263K↓R1264
GGSIQQYIYK	1264R↓G1265
QEPVQKCPGR	1274K↓Q1275
LAYQDK	1324R↓L1325
VSILWR	1338K↓V1339
KPDGYDYYAFSK	1360R↓K1361
DQYYNIDVPSR	1372K↓D1373
VWYNCP	1398K↓V1399
24 hours	
CGEGYSR	33R↓C34
RVCTAELSCK	61K↓R62
GRECDCDAQCK	80R↓E81
KAPPPSGASQTIK	117K↓K118
KVIESEEITEEHSVSENQESSSSSSSSSSSTIR	146K↓K147
VIESEEITEEHSVSENQESSSSSSSSSSSTIR	147K↓V148
VTTPDTSTTQHNK	232K↓V233
ETSLTVNK	272K↓E273
ETTVETK	280K↓E281
ETTTTNK	287K↓E288
QTSTDGKEKTTSAK	294K↓Q295
TTSAKETQSIEK	303K↓T304
ETQSIEK	308K↓E309
DLAPTSK	319K↓D320
EPAPTTTK	394K↓E395
ELAPTTTK	731K↓E732
EPAPTTPKEPAPTTPK	832K↓E833
ALENSPK	887K↓A888
LVEVNPK	1086K↓L1087
CNCEGK	1201R↓C1202
DSQYWR	1212K↓D1213
FTNDIK	1218R↓F1219
DAGYPKPIFK	1224K↓D1225
RGGSIQQYIYK	1263K↓R1264
GGSIQQYIYKQEPVQK	1264R↓G1265
QEPVQK	1274K↓Q1275
IQYSPAR	1317R↓I1318
GVLHNEVK	1330K↓G1331
KPDGYDYYAFSK	1360R↓K1361
DQYYNIDVPSR	1372K↓D1373
SGQTLSK	1391R↓S1392

Supplementary Figure 1: **a)** Silver-stained 10% SDS-PAGE gel of *in vitro* cleavage of PRG4 by tryptase ß (1:100 enzyme/substrate), magenta arrow indicates cleaved PRG4. A deglycosylated enzyme was added to PRG4 before the addition of tryptase (1:100 enzyme/substrate), which resulted in degradation of PRG4. This is a representative image of an experiment that was repeated three times with similar results. **b)** Schematic of the ATOMS workflow created with BioRender. PRG4 was incubated for 18 hrs before being subjected to formaldehyde labeling (+28 Da). Tryptase ß and PRG4 were incubated for 18 hrs before being subjected with deuterated formaldehyde (+34 Da). Both samples were mixed and trypsin was added before being analyzed on an LC-MS/MS. RAW data were analyzed by MaxQuant. **c)** Different cleavage sites were identified between the different samples at 1 h and 24 hrs comparing heavy dimethylated sample (PRG4) to the light dimethylated sample (tryptase ß + PRG4).

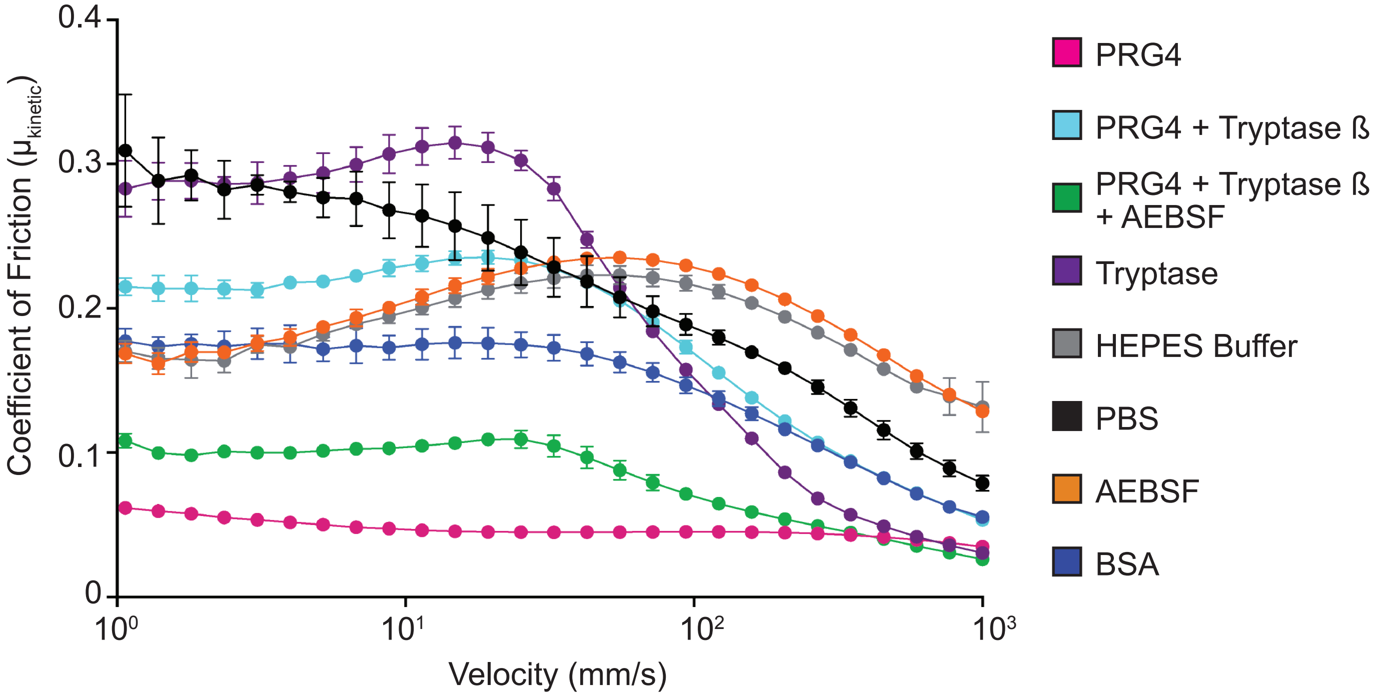

Supplementary Figure 2: Lubrication of PBS buffer (black), HEPES buffer (orange), tryptase (grey), PRG4 (blue), PRG4 + tryptase incubated for 18 h (1:100 enzyme/substrate) (magenta), PRG4 + tryptase + 1 mM AEBSF incubated for 18 h (1:100 enzyme/substrate) (green). The error bars denote the error of the mean ± standard deviation (SD) as obtained from 5 independent experiments.

Supplementary Figure 3

Das *et al.*

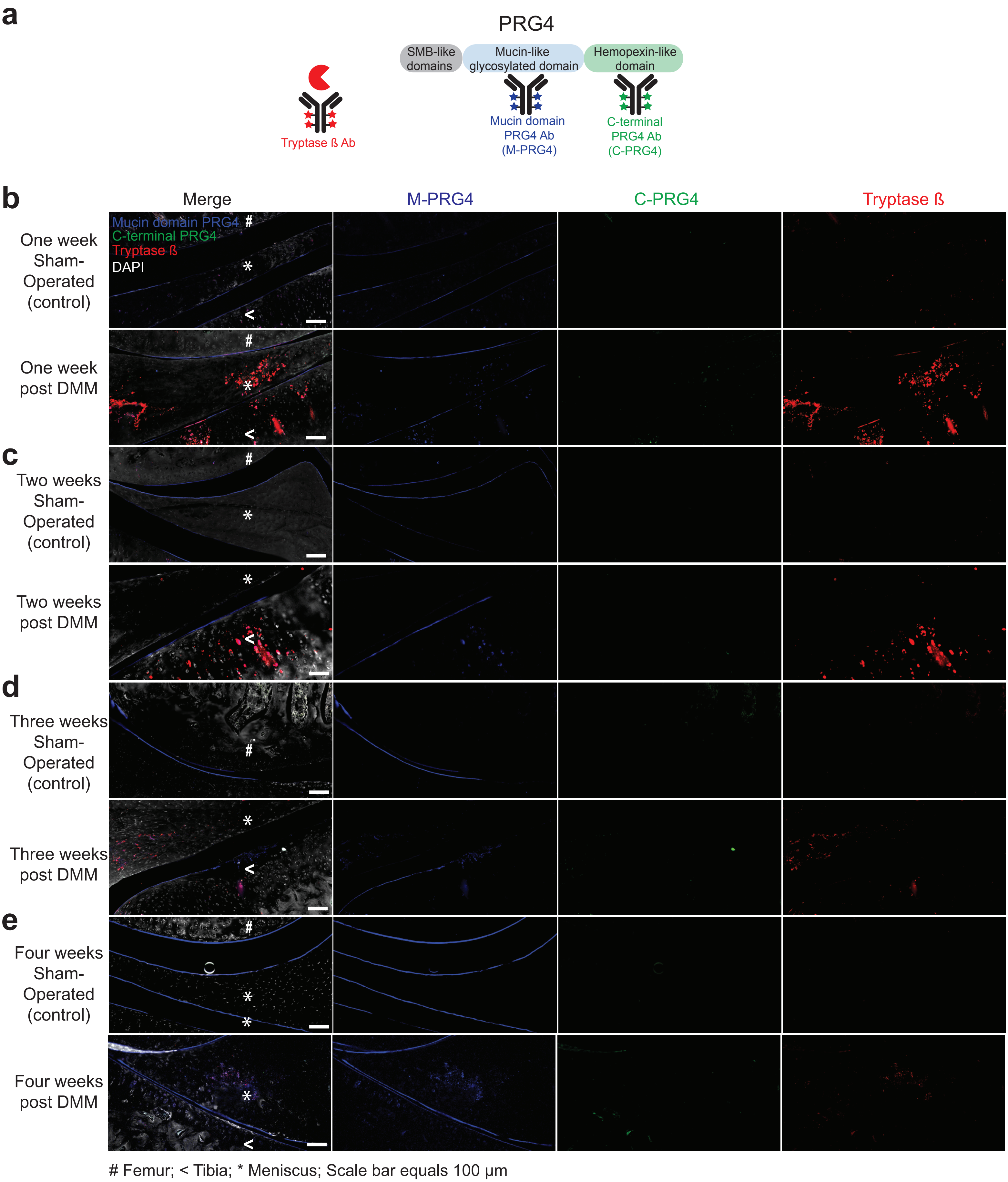

Supplementary Figure 3: **a)** Schematic of the antibodies used: mucin domain of PRG4 in blue, C-terminal domain of PRG4 in green and tryptase ß in red (created with BioRender). PRG4 and tryptase ß staining in rat joints **b)** one week, **c)** two weeks, **d)** three weeks and **e)** four weeks post-DMM. Sham-operated surgery controls are included for each time point. (n = 15 rats in total, n = 3 per groups). Scale bar = 100 µm. # indicates femur, < indicates tibia and * indicates the meniscus.

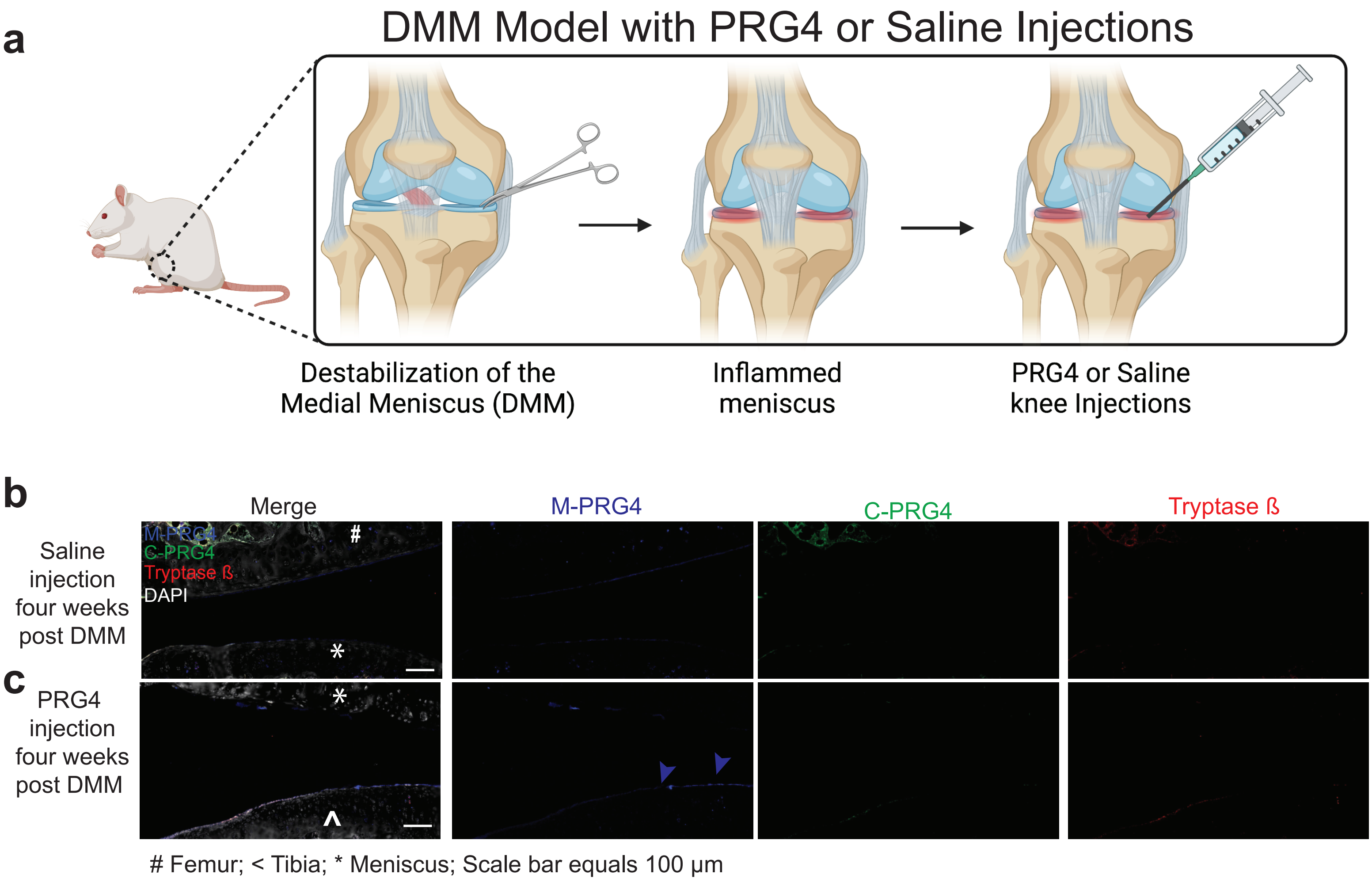

Supplementary Figure 4: **a)** Schematic of the experimental workflow of saline and PRG4 injection in the DMM model (created with BioRender). PRG4 and tryptase ß staining in rat joints four weeks post DMM in **b)** saline- or **c)** PRG4-injected animals. (n = 3 per group). Scale bar = 100 µm. # indicates femur, < indicates tibia and * indicates the meniscus.

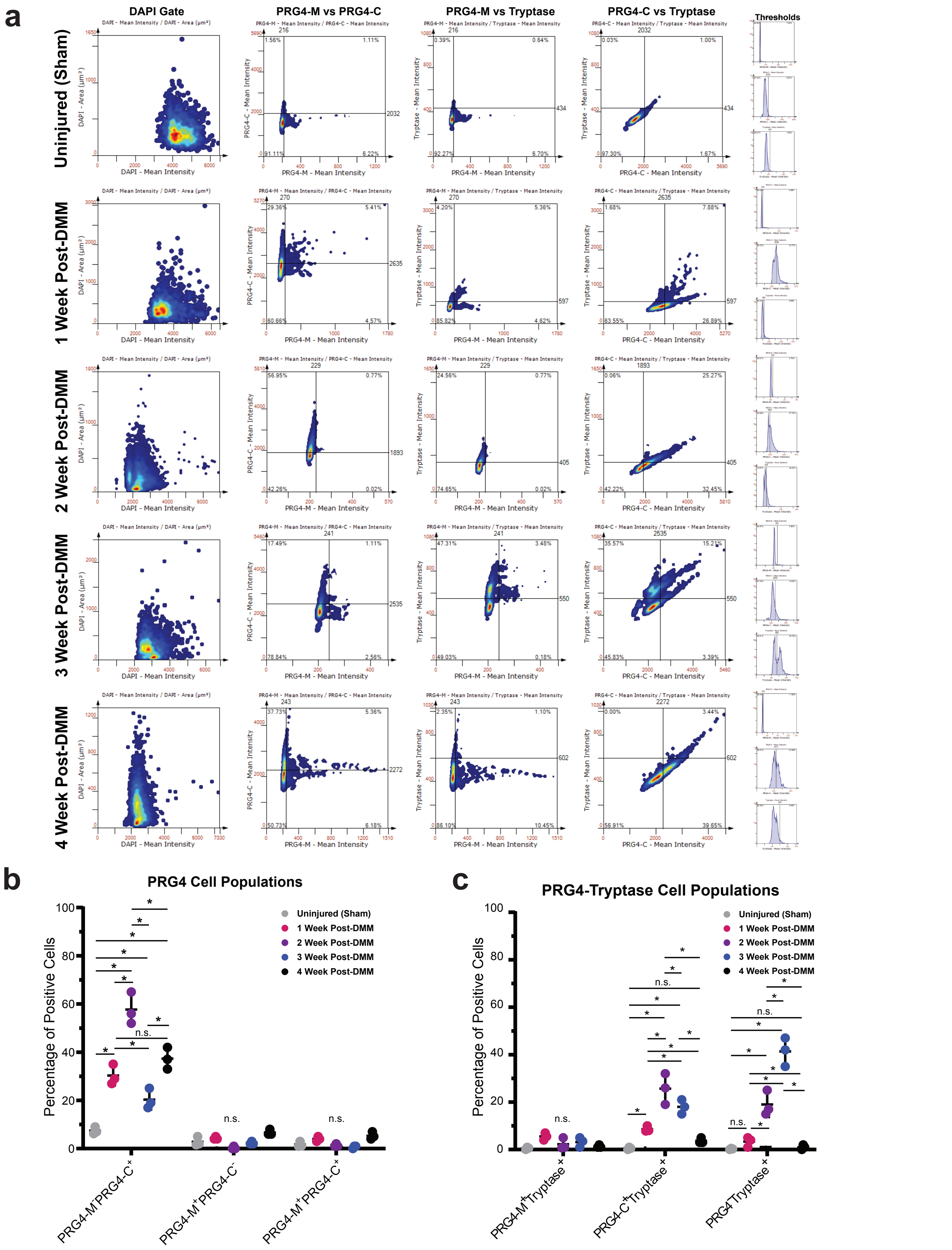

Supplementary Figure 5: Cells of a given phenotype were identified and quantitated using the TissueQuest software (TissueGnostics), with cut-off values determined relative to the negative controls (non-stained and secondary alone controls). **a)** Gating and quantification of single/double positive cells were undertaken using these thresholds. **b)** Quantification of PRG4 cell populations. **c)** Quantification of Tryptase and PRG4 cell populations. Statistical analysis was determined by a two-tailed unpaired Student's t test. *p < 0.05, **, p < 0.01, ***, p < 0.001, and n.s., not significant. The error bars denote the error of the mean ± standard deviation (SD) as obtained from from 3 independent experiments.

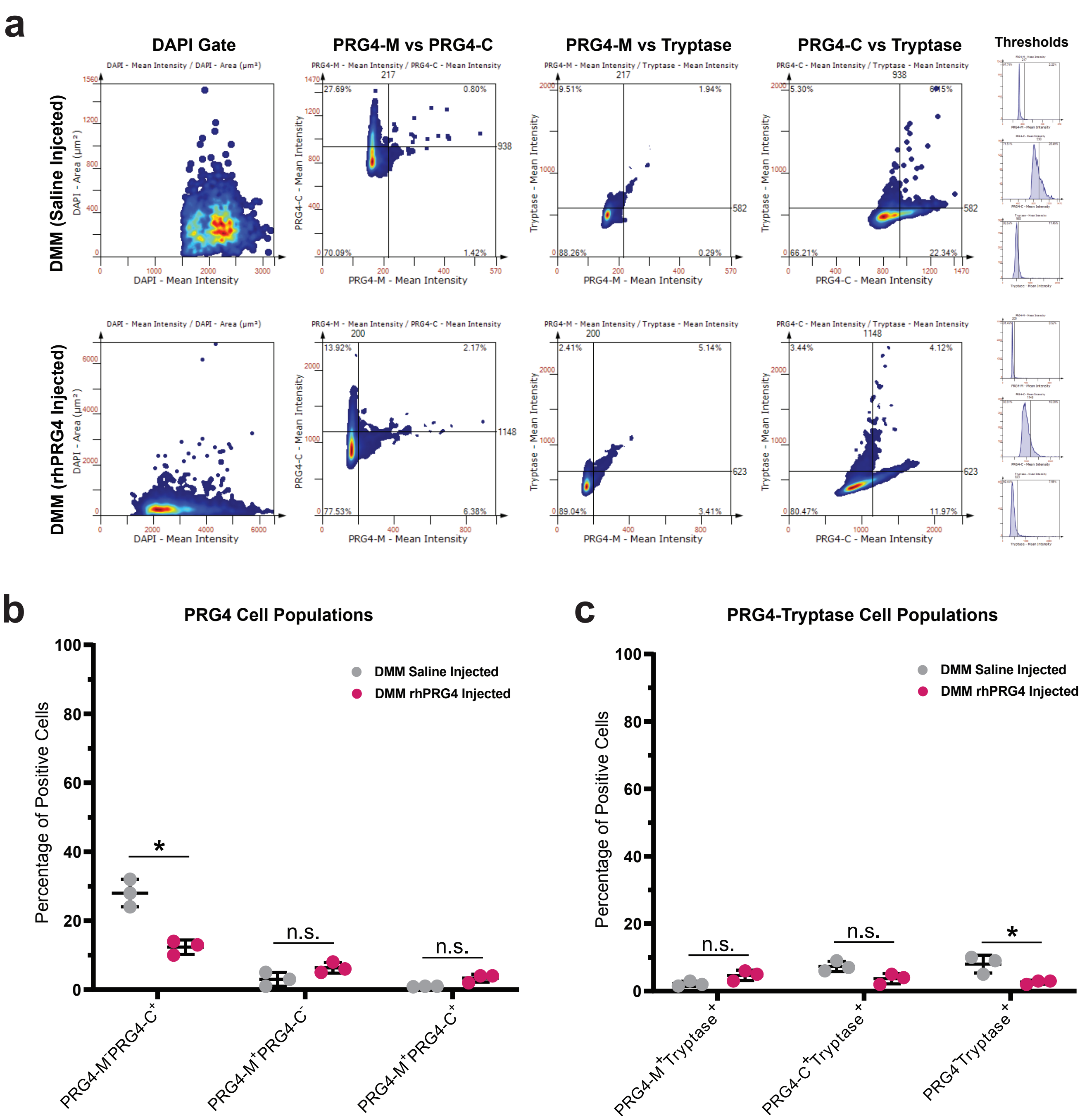

Supplementary Figure 6: Cells of a given phenotype were identified and quantitated using the TissueQuest software (TissueGnostics), with cut-off values determined relative to the negative controls (non-stained and secondary alone controls). **a)** Gating and quantification of single/double positive cells were undertaken using these thresholds. **b)** Quantification of PRG4 cell populations. **c)** Quantification of Tryptase and PRG4 cell populations. Statistical analysis was determined by a two-tailed unpaired Student's t test. *p < 0.05, **, p < 0.01, ***, p < 0.001, and n.s., not significant. The error bars denote the error of the mean ± standard deviation (SD) as obtained from from 3 independent experiments.

a

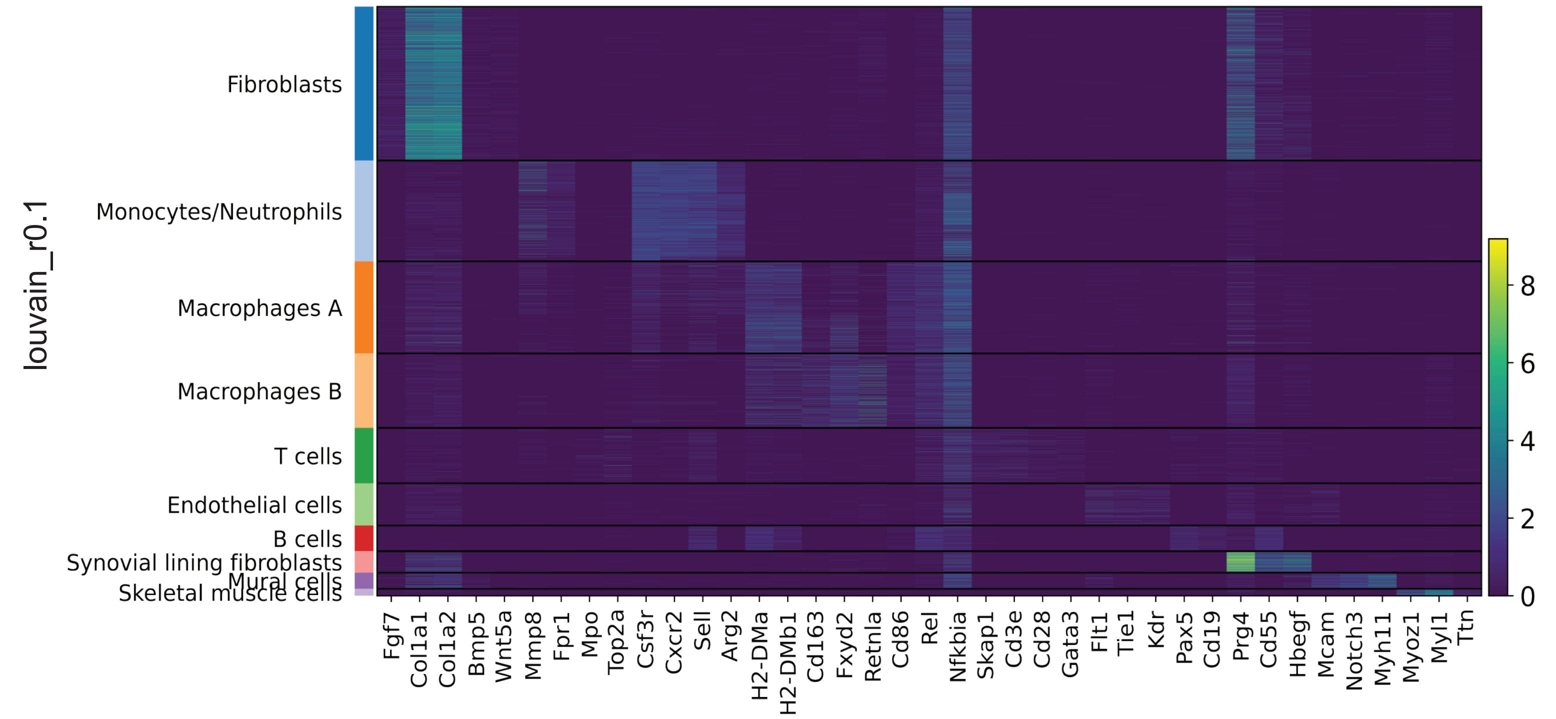

b

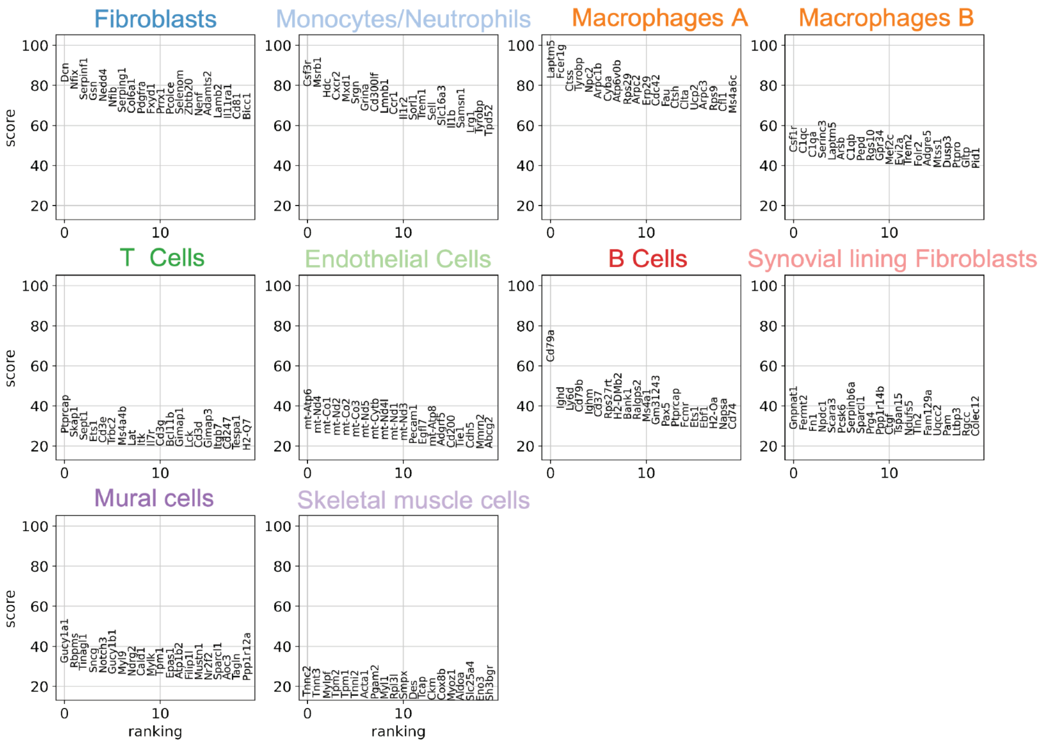

Clusters	Cell name	**Cell markers used for annotation** (Muench DE et al. (2022) *Journal of* Immunology **208**(6): 1315-1328 and https://www.proteinatlas.org/)	Counts
0	Fibroblasts	Fgf7, Col1a1, Col1a2, Bmp5, Wnt5a, Rcn3, Col6a1, Col6a2, Col1a2, Cthrc1, Lum, Fkbp10, Epyc, Tmem119, Olfml3, Copz2, Loxl1, Col5a2, Col5a1, Lum, Fbln1, Fbln2, Cd34, Pdgfra	6399
1	Monocytes/Neutrophils	Csf3r, Cxcr2, Sell, Arg2, Cd14, Mmp8, Fpr1, Mpo, Tgm3, Mmp9, Cd300c, Hk3, Ccr1, Cd300lf, Lilra6, Igsf6, Fcer1g, Tyrobp	4191
2	Macrophages A	Cx3cr1, Retnla, Cxcl2, Tnf, Ciita, Cd38, Adgre1, Mrc1, Cd86, Cd68, Fcgr1, Fcgr2b, Cd163, Arg1, Cd36	3827
3	Macrophages B		3098
4	T cells	Skap1, Cd3e, Cd28, Gata3, Trac, Cd5, Cd2, Cd4, Cd8a, Icos, Gzmb, Gzma, Cd40lg	2302
5	Endothelial Cells	Flt1, Tie1, Kdr, Cdh5, Sele, Vwf, Angpt2, Pecam1	1753
6	B Cells	Pax5, Cd19, Vpreb3, Pax5, Bank1, Fcrl1, Cr2, Ms4a1, Blk, Cd74, Cd79a, Iglc1, Ighd, Ighm	1070
7	Synovial lining fibroblasts	Prg4, Cd55, Hbegf	895
8	Mural Cells	Mcam, Notch3, Myh11, Des, Tagln	665
9	Skeletal Muscle Cells	Myoz1, Myl1, Ttn, Myod1, Tpm1, Acta1	296

Supplementary Figure 7: **Cell identification, annotation, and proportion based on single-cell RNA sequencing data. a)** Heat map of differential gene expression defined by the original paper in scRNA-seq data. **b)** Marker genes were obtained from the original work and the protein atlas website. Calculation of a correlation matrix for genes in each cluster versus other clusters (scanpy.pl.rank_genes_groups.) genes that are up-regulated in the cluster will be compared to all other clusters (marker genes). Our new analysis included 24,496 cells from 5 mice.

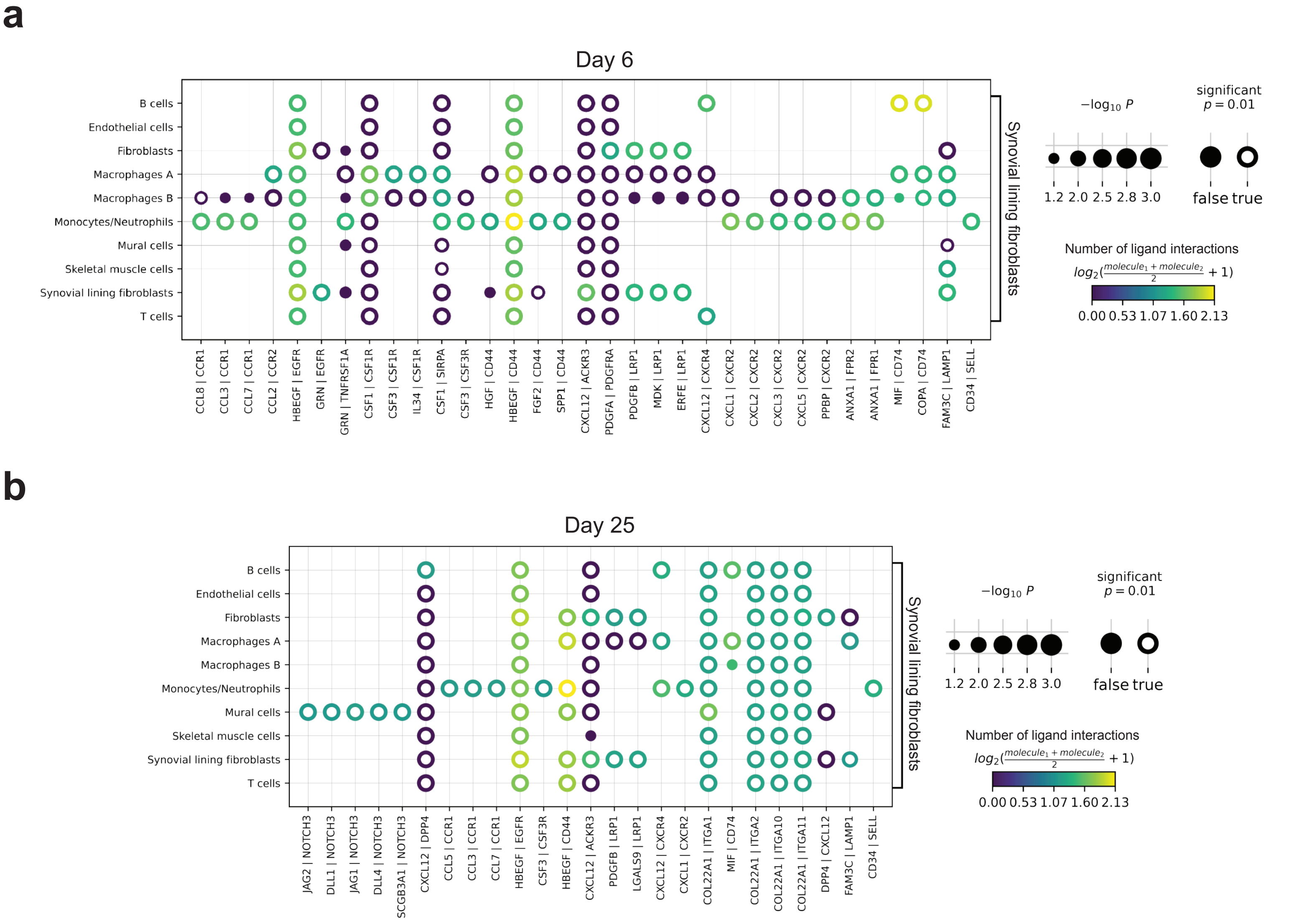

Supplementary Figure 8: **Cell-cell interaction via ligand interactions.** Analysis using the CellPhoneDB method of the crosstalk between the synovial lining fibroblasts in the synovial fluid of **a)** Day 6 and **b)** Day 25 groups. For **a)** and **b)**, *p*-values are computed from one-sided permutation test. Adjustments were made for multiple comparisons.

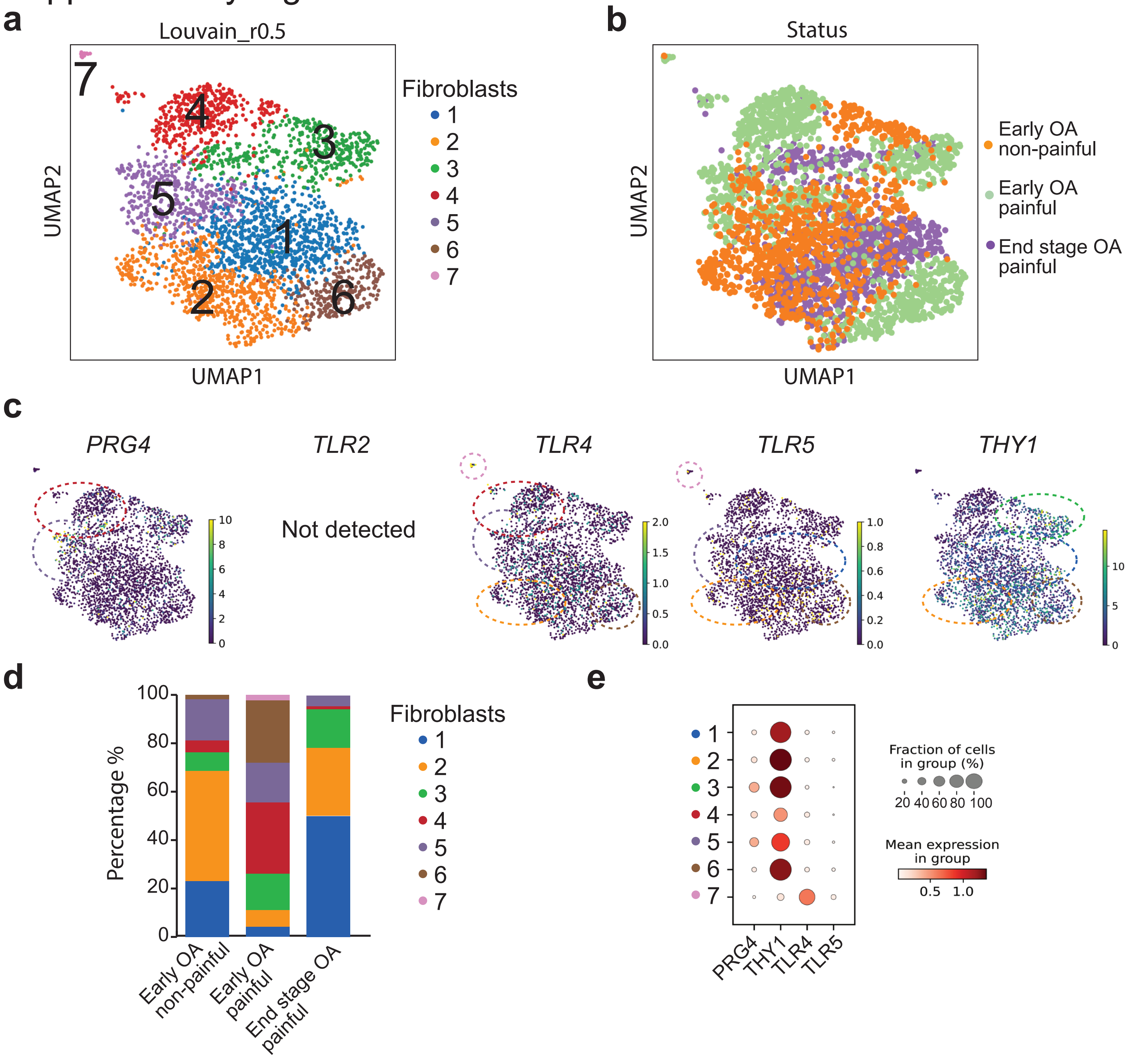

Supplementary Figure 9: **Single-cell RNA-sequencing analysis of human OA fibroblasts (GSE176308).** **a)** Louvain clustering and cell annotation. Using Louvain clustering, we distinguished 7 cell clusters in the human OA fibroblasts with a resolution of 0.5 (n = 3). **b)** Classification included cells based on the disease status, including early OA non-painful, early OA painful and end stage OA painful. **c)** The expression pattern of *PRG4*, *TLR4*, *TLR5*, and *THY1* in all clusters using UMAP. **d)** Population distribution in percentages of scRNA-seq data of OA fibroblats isolated at the indicated conditions. **e)** The expression pattern of *PRG4*, *TLR4*, *TLR5*, and *THY1* genes in the OA fibroblasts.

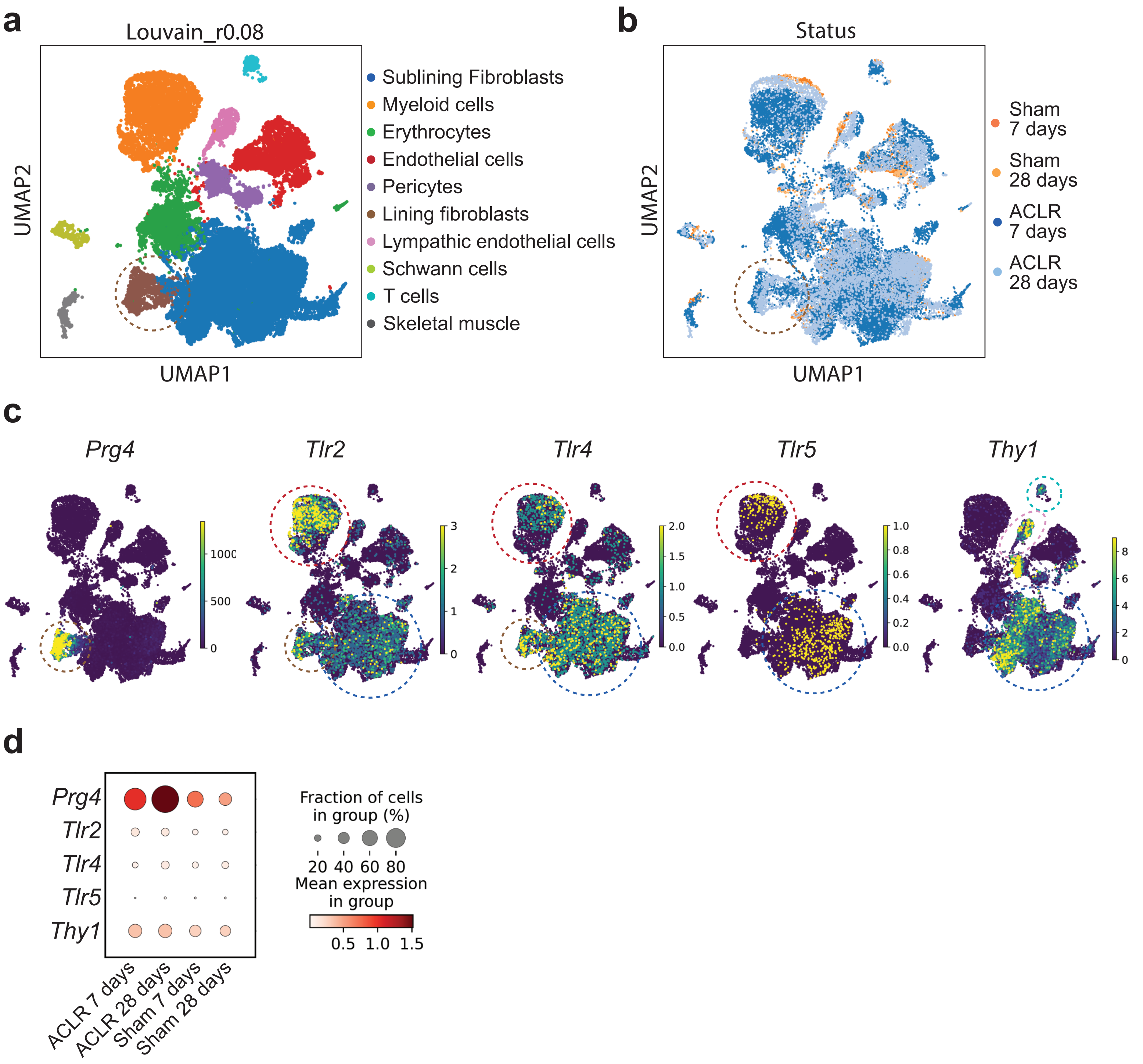

Supplementary Figure 10: **Single-cell RNA-sequencing analysis of murine knee synovium (GSE211584).** **a)** Louvain clustering and cell annotation. Using Louvain clustering, we distinguished 10 cell clusters of murine knee synovium with a resolution of 0.08 (n = 6). **b)** Classification included cells based on the model disease status, including sham surgery at 7 days, sham surgery at 28 days, anterior cruciate ligament rupture-based model of joint injury (ACLR) at 7 and 28 days. **c)**The expression pattern of *Prg4*, *Tlr2*,*Tlr4*,*Tlr5*, and*Thy1* in all clusters using UMAP. **d)** The expression pattern of *Prg4*, *Tlr2*,*Tlr4*,*Tlr5*, and*Thy1* genes in the synovium of ACLR model.

a

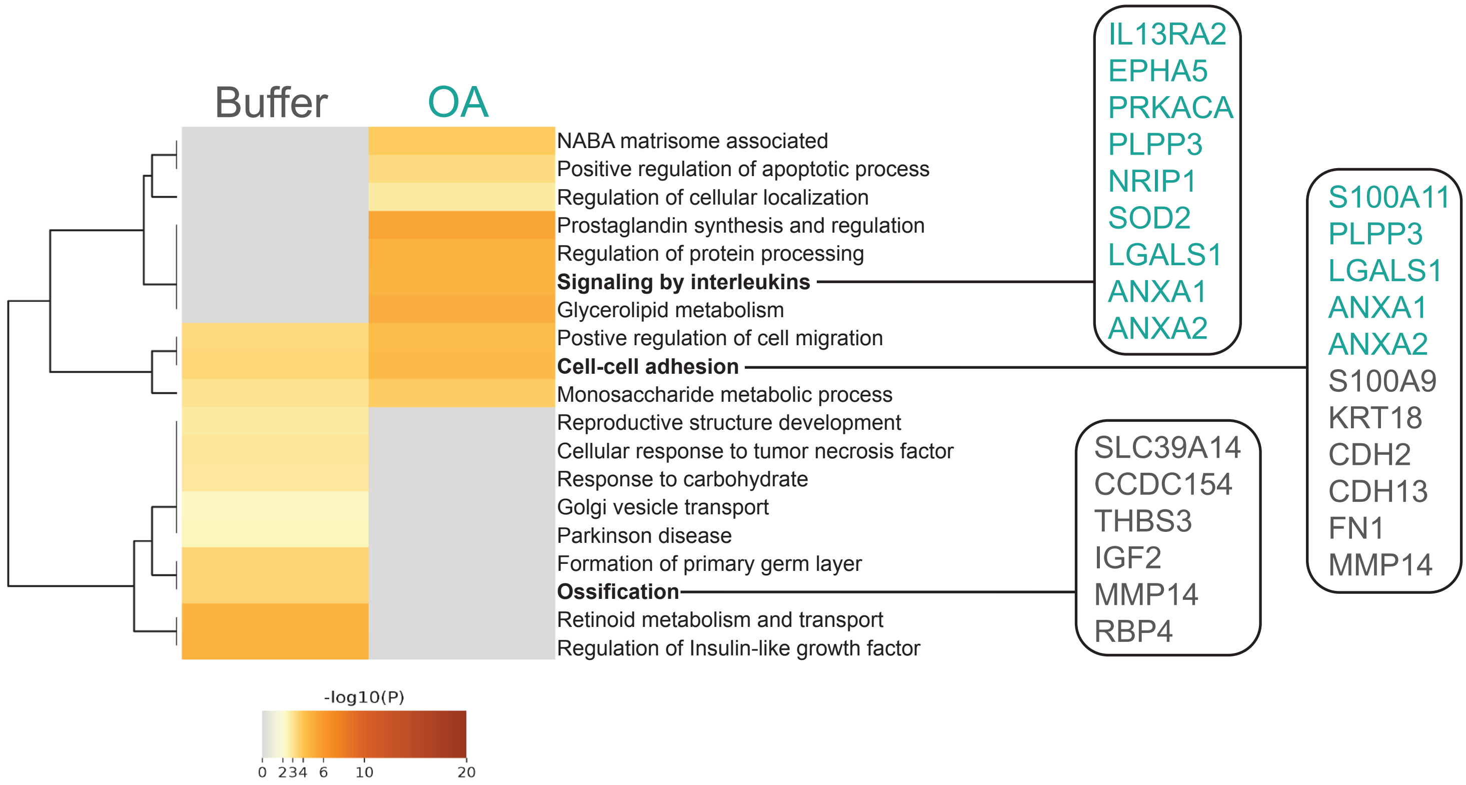

b

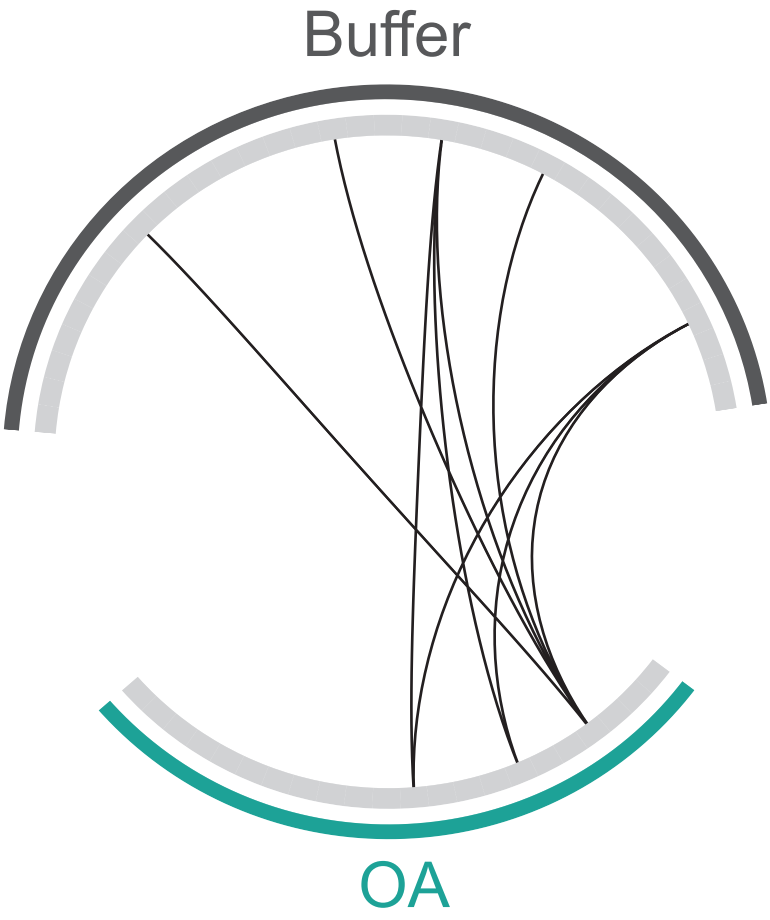

Supplementary Figure 11: **a)** Heatmap of statistically enriched terms in which accumulative hypergeometric p values and enrichment factors were calculated and used for filtering as performed as a two-sided analysis. The remaining significant terms were hierarchically clustered into a tree based on kappa-statistical similarities among their gene memberships. A 0.3 kappa score was applied as the threshold to cast the tree into term clusters. Dendrogram displays selected terms with the best p value within each cluster as its representative term. Heatmap cells are colored by their p values, and gray cells indicate the lack of enrichment for that term in the corresponding gene list. **b)** Circos plot indicating the overlapping genes between non-OA buffer treated fibrobasts and OA fibroblasts. Data analyzed from 3 biological replicates and 2 technical replicates.

a

Patient number	Joint	Age	Sex
1	Right	75	Male
2	Left	59	Male
3	Left	74	Male

b

TAILS data
Overlap Analysis

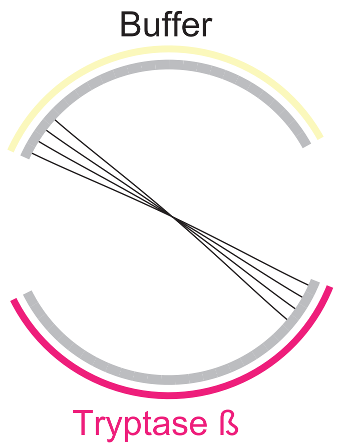

c

preTAILS data
Protein-protein Interaction Networks

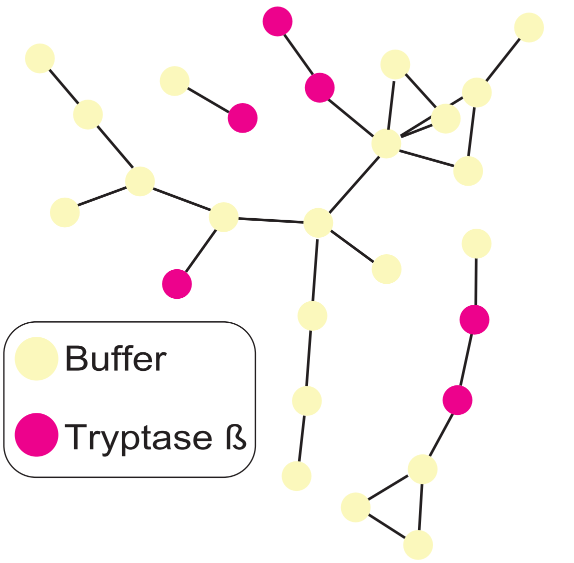

d

preTAILS data
Overlap Analysis

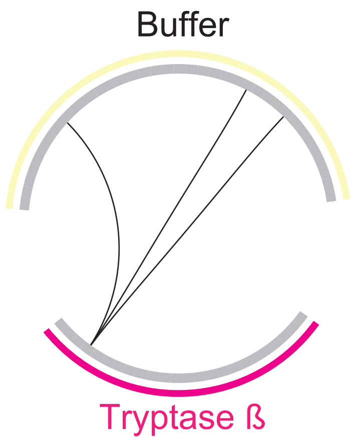

e

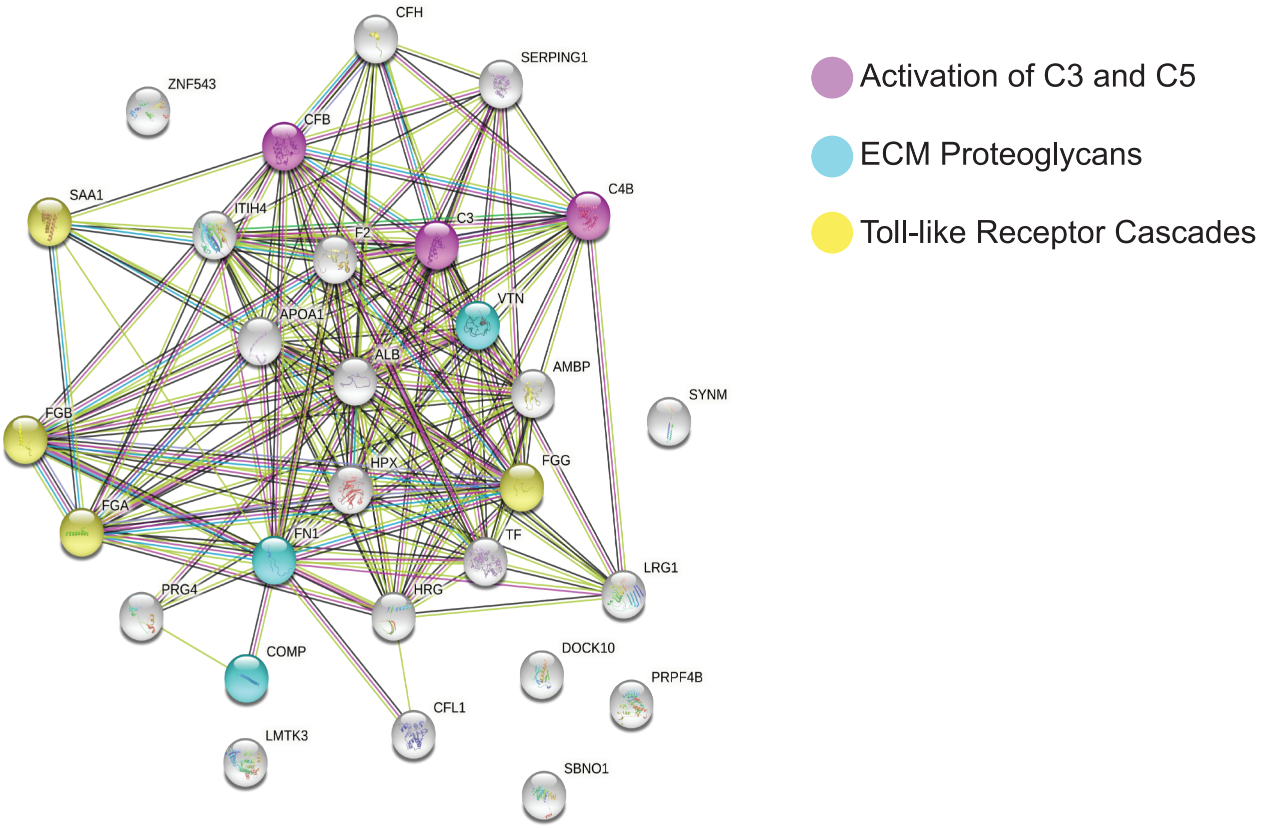

Supplementary Figure 12: **a)** Patients information about the synovial fluid used for N-terminomics (n =3 per group). **b)** Circos plot indicating the overlapping proteins between buffer and tryptase ß-treated synovial fluid TAILS data. **c)** Protein–protein interactions (PPIs) among each input gene list were extracted from PPI data source and formed a PPI network **d)** Circos plot indicating the overlapping proteins between buffer and tryptase ß-treated synovial fluid preTAILS data. **e)** STRING Analysis of Quantitative Proteomics Data of buffer-treated synovial fluid. Analysis of protein−protein interaction network by STRING v11. Elevated proteins from buffer-treated samples identified from the quantitative proteomics analysis were mapped by searching the STRING v11 software with a confidence level of 1% false discovery rate. Colored lines between the proteinsindicate different types of interaction evidence: known interactions (teal), experimentally determined (pink), predicted interactions gene neighborhood (green), gene fusions (red), gene co-occurrence (blue), text-mining (yellow), coexpression (black), protein homology (purple).

a

Healthy			
**Patient number**	**Joint**	**Age**	**Sex**
1	Right	75	Male
2	Left	59	Male
3	Left	74	Male

OA				
**Patient number**	**Joint**	**Stage**	**Age**	**Sex**
4	Left	KL1/2	31	Male
5	Left	KL1/2	47	Male
6	Right	KL3/4	62	Male

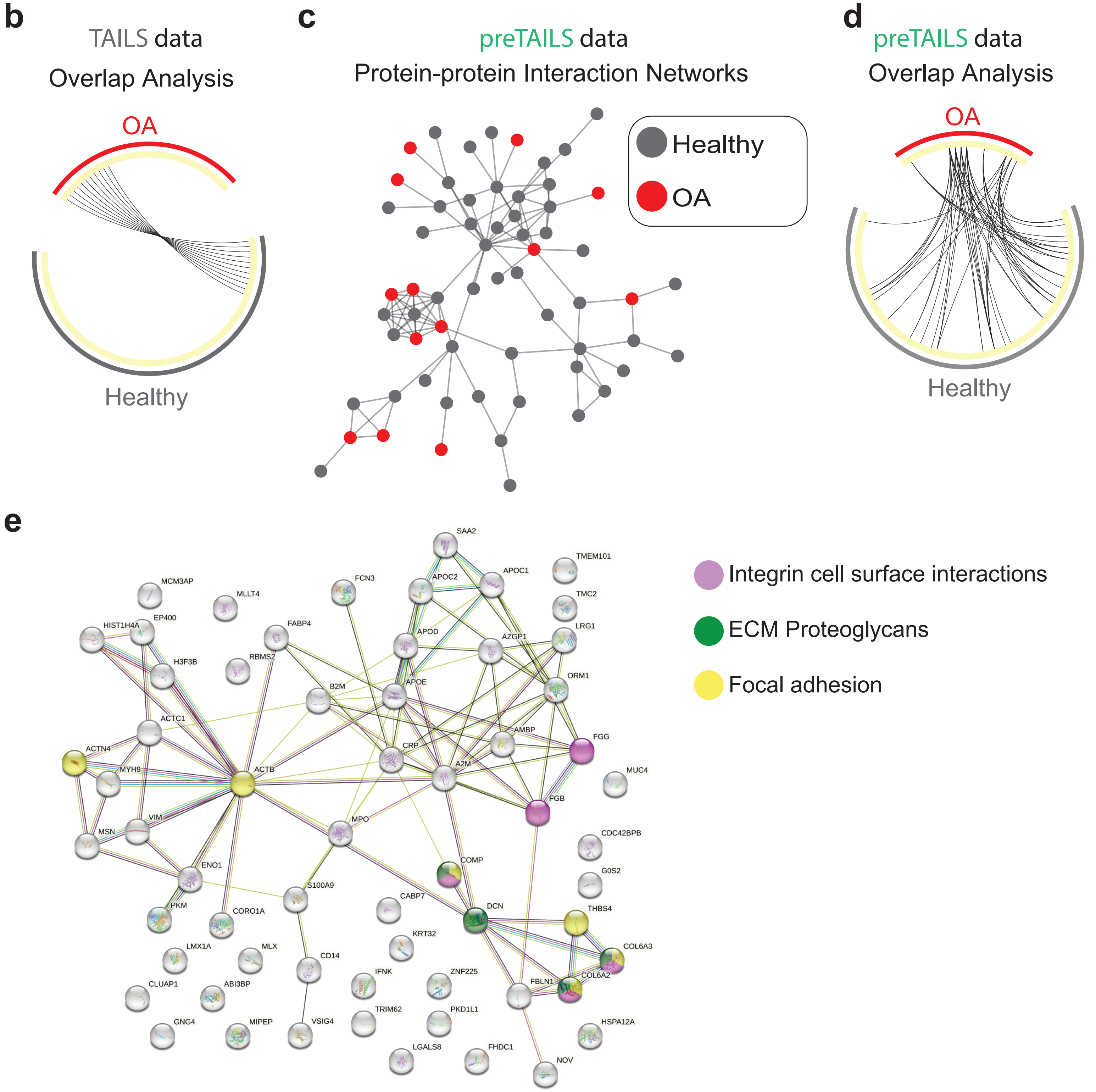

Supplementary Figure 13: **a)** Patients information about the synovial fluid used for N-terminomics (n = 3 per group). **b)** Circos plot indicating the overlapping proteins between OA patients and healthy synovial fluid TAILS data. **c)** Protein–protein interactions (PPIs) among each input gene list were extracted from PPI data source and formed a PPI network **d)** Circos plot indicating the overlapping proteins between OA patients and healthy synovial fluid preTAILS data. **e)** STRING Analysis of Quantitative Proteomics Data of healthy synovial fluid. Analysis of protein−protein interaction network by STRING v11. Elevated proteins from buffer-treated samples identified from the quantitative proteomics analysis were mapped by searching the STRING v11 software with a confidence level of 1% false discovery rate. Colored lines between the proteins indicate different types of interaction evidence: known interactions (teal), experimentally determined (pink), predicted interactions gene neighborhood (green), gene fusions (red), gene co-occurrence (blue), text-mining (yellow), coexpression (black), protein homology (purple).
