# Supplementary material for: Tryptase β regulation of joint lubrication and inflammation via proteoglycan-4 in osteoarthritis
Source: Nat Commun. 2023 Apr 6;14:1910. doi: 10.1038/s41467-023-37598-3 (PMC10079686; doi:10.1038/s41467-023-37598-3)
Supplement: Supplementary file 3 — Reporting Summary [file 41467_2023_37598_MOESM3_ESM.pdf]

nature portfolio

Corresponding author(s): Antoine Dufour

Last updated by author(s): Mar 5, 2023

Reporting Summary

Nature Portfolio wishes to improve the reproducibility of the work that we publish. This form provides structure for consistency and transparency in reporting. For further information on Nature Portfolio policies, see our Editorial Policies and the Editorial Policy Checklist.

Statistics

For all statistical analyses, confirm that the following items are present in the figure legend, table legend, main text, or Methods section.

n/a	Confirmed	
☐	☒	The exact sample size (*n*) for each experimental group/condition, given as a discrete number and unit of measurement
☐	☒	A statement on whether measurements were taken from distinct samples or whether the same sample was measured repeatedly
☐	☒	The statistical test(s) used AND whether they are one- or two-sided<br>*Only common tests should be described solely by name; describe more complex techniques in the Methods section.*
☐	☒	A description of all covariates tested
☐	☒	A description of any assumptions or corrections, such as tests of normality and adjustment for multiple comparisons
☐	☒	A full description of the statistical parameters including central tendency (e.g. means) or other basic estimates (e.g. regression coefficient) AND variation (e.g. standard deviation) or associated estimates of uncertainty (e.g. confidence intervals)
☐	☒	For null hypothesis testing, the test statistic (e.g. *F*, *t*, *r*) with confidence intervals, effect sizes, degrees of freedom and *P* value noted<br>*Give P values as exact values whenever suitable.*
☐	☒	For Bayesian analysis, information on the choice of priors and Markov chain Monte Carlo settings
☐	☒	For hierarchical and complex designs, identification of the appropriate level for tests and full reporting of outcomes
☐	☒	Estimates of effect sizes (e.g. Cohen's *d*, Pearson's *r*), indicating how they were calculated

Our web collection on statistics for biologists contains articles on many of the points above.

Software and code

Policy information about availability of computer code

Data collection	Data collection was performed on an Orbitrap Fusion Lumos Tribrid mass spectrometer (Thermo Fisher Scientific, Mississauga, ON) operated with Xcalibur (version 4.0.21.10) and coupled to a Thermo Scientific Easy-nLC (nanoflow Liquid Chromatography) 1,200 system. Also, the tribology test was done with a glass-polydimethylsiloxane (PDMS) polymer interface using a ball-on-cylinder geometry on a MCR 302 rate controlled rotational rheometer equipped with a tribology unit (Anton Paar, Graz, Austria). TissueQuest v7.1 software (TissueGnostics) was used to quantify histology experiments. All additional details are included in the material and methods.
Data analysis	Data was analyzed with MaxQuant (v.l.6.0.1), MSstatsTMT (v2.4.0), R software (v4.2.0), TopFIND, TopFINDer, Uniprot, MEROPS, STRING.vll, Metascape, GraphPad Prism version 9, TissueQuest v7.1 software (TissueGnostics) and Microsoft Excel. All additional details are included in the material and methods.

For manuscripts utilizing custom algorithms or software that are central to the research but not yet described in published literature, software must be made available to editors and reviewers. We strongly encourage code deposition in a community repository (e.g. GitHub). See the Nature Portfolio guidelines for submitting code & software for further information.

Data

Policy information about availability of data

All manuscripts must include a data availability statement. This statement should provide the following information, where applicable:

- Accession codes, unique identifiers, or web links for publicly available datasets
- A description of any restrictions on data availability
- For clinical datasets or third party data, please ensure that the statement adheres to our policy

All data generated or analysed during this study are included in this published article (and its supplementary information files). Proteomics RAW data were deposited to ProteomeXchange via the Proteomics Identification Database (PRIDE) under accession number PXD037040. All data about identified and quantified peptides and proteins are included in the Supplementary Tables 1-11. Gene Expression Omnibus accession numbers: GSE184609, GSE176308, and GSE211584. Source data are provided with this paper.

Human research participants

Policy information about studies involving human research participants and Sex and Gender in Research.

Reporting on sex and gender	For human samples, all samples used were male.
Population characteristics	All human samples used were from male patients with an age range between 31 and 75 years old.
Recruitment	Healthy Group (n = 7): Criteria for control cadaveric donations were an age of 40 years or older, no history of arthritis, joint injury, or surgery (including visual inspection of the cartilage surfaces during recovery), no prescription anti-inflammatory medications, no co-morbidities (such as. diabetes/cancer), and availability within 4 h of death. OA Group (n = 3): Criteria were an age of 40 years or older, OA diagnosed based on the American College of Rheumatology criteria with X-ray documentation, and no evidence of autoimmune disease or RA. Synovial biopsies. from the medial compartment were collected during routine arthroscopy. Only patients with an Outerbridge score of 3 or greater were selected for this study.
Ethics oversight	Our research complies with all relevant ethical regulations and the University of Calgary Research Ethics Board approved this study protocol. Our human studies were carried out in adherence to the principles of the Declaration of Helsinki. Informed consent to participate was obtained by written agreement. We obtained consent to publish information that identifies the age, sex, gender, and stage of disease. The study protocol was approved by the University of Calgary Research Ethics Board. All methods were carried out in accordance with the approved guidelines.

Note that full information on the approval of the study protocol must also be provided in the manuscript.

Field-specific reporting

Please select the one below that is the best fit for your research. If you are not sure, read the appropriate sections before making your selection.

[x] Life sciences [ ] Behavioural & social sciences [ ] Ecological, evolutionary & environmental sciences

For a reference copy of the document with all sections, see nature.com/documents/nr-reporting-summary-flat.pdf

Life sciences study design

All studies must disclose on these points even when the disclosure is negative.

Sample size	Samples size for the shotgun proteomics and N-terminomics experiments were determined as per previous studies and datasets that were analyzed (Gordon et al. (2019) ACS chemical biology 14(11):2471-2483).
Data exclusions	No data were excluded.
Replication	All experiments were repeated at least once and were successful.
Randomization	No need for randomization as all samples were included in our analysis and are described as individual data point.
Blinding	Histology experiments performed on rat joints from the DMM model were blinded.

Reporting for specific materials, systems and methods

We require information from authors about some types of materials, experimental systems and methods used in many studies. Here, indicate whether each material, system or method listed is relevant to your study. If you are not sure if a list item applies to your research, read the appropriate section before selecting a response.

Materials & experimental systems

n/a	Involved in the study	
☐	☒	Antibodies
☐	☒	Eukaryotic cell lines
☒	☐	Palaeontology and archaeology
☐	☒	Animals and other organisms
☐	☒	Clinical data
☒	☐	Dual use research of concern

Methods

n/a	Involved in the study	
☒	☐	ChIP-seq
☐	☒	Flow cytometry
☒	☐	MRI-based neuroimaging

Antibodies

Antibodies used	CD90 (Clone# 5El0, PE), CD68 (clone# Yl/82A, FITC), cell viability marker was FVS510 (BV510) were from BD Biosciences, Mississauga. Primary antibody for PRG4; mucin domain antibody 9G3 (Millipore, MABT401) bound to Dylight 630 (Abeam Millipore, ab201803) and C-terminal antibody, LPN (lnvitrogen, PA3-118) bound to Dylight 488 (Abeam Millipore, ab201799) or beta-tryptase (Biolegend, 369402) bound to Dylight 550 (Abeam Millipore, ab201800) were used.
Validation	All antibodies were used as the manufacturer's recommendation or from previous publications.

Eukaryotic cell lines

Policy information about cell lines and Sex and Gender in Research

Cell line source(s)	Human TLRnull, TLR2+, TLR4+ and TLR5+ cell lines (lnvivogen, San Diego, CA) were cultured as directed by the manufacturer.
Authentication	Cell lines were authenticated by the manufacturer (lnvivogen, San Diego, CA) upon arrival.
Mycoplasma contamination	All cells were tested for mycoplasma and were found to be negative.
Commonly misidentified lines (See ICLAC register)	No cells from that list were used in this publication.

Animals and other research organisms

Policy information about studies involving animals; ARRIVE guidelines recommended for reporting animal research, and Sex and Gender in Research

Laboratory animals	8 female and 5 male 10-week-old lewis rats were used.
Wild animals	No wild animals were used.
Reporting on sex	8 female and 5 male 10-week-old lewis rats were used.
Field-collected samples	Not applicable.
Ethics oversight	The study protocol was approved by the University of Calgary Research Ethics Board. All methods were carried out in accordance with the approved guidelines. Animal studies were carried out in accordance with the recommendations in the Canadian Council on Animal Care Guidelines. Animal protocols and surgical procedures in this study were approved by the University of Calgary Health Sciences Animal Care Committee.

Note that full information on the approval of the study protocol must also be provided in the manuscript.

Clinical data

Policy information about clinical studies

All manuscripts should comply with the ICMJEguidelines for publication of clinical research and a completedCONSORT checklist must be included with all submissions.

Clinical trial registration	Not applicable.
Study protocol	Not applicable.
Data collection	Not applicable.
Outcomes	Not applicable.

Flow Cytometry

Plots

Confirm that:

- [x] The axis labels state the marker and fluorochrome used (e.g. CD4-FITC).
- [x] The axis scales are clearly visible. Include numbers along axes only for bottom left plot of group (a 'group' is an analysis of identical markers).
- [x] All plots are contour plots with outliers or pseudocolor plots.
- [x] A numerical value for number of cells or percentage (with statistics) is provided.

Methodology

Sample preparation	Not applicable.
Instrument	Not applicable.
Software	Not applicable.
Cell population abundance	Not applicable.
Gating strategy	Not applicable.

- [ ] Tick this box to confirm that a figure exemplifying the gating strategy is provided in the Supplementary Information.
