# Supplementary material for: Tryptase β regulation of joint lubrication and inflammation via proteoglycan-4 in osteoarthritis
Source: Nat Commun. 2023 Apr 6;14:1910. doi: 10.1038/s41467-023-37598-3 (PMC10079686; doi:10.1038/s41467-023-37598-3)

Source Data File

Tryptase β regulation of joint lubrication and inflammation via proteoglycan-4 in osteoarthritis

Nabangshu Das[1,2,3,4], Luiz G. N. de Almeida[2,3,4,5], Afshin Derakhshani[2,3,4,5], Daniel Young[2,3,4,5], Kobra Mehdinejadiani[2,3,4,5], Paul Salo[3], Alexander Rezansoff[1,3], Gregory D Jay[6], Christian P. Sommerhoff[7], Tannin A. Schmidt[3,8], Roman Krawetz[3,9*], Antoine Dufour[1,2,3,4,5,10*]

[1]Faculty of Kinesiology, University of Calgary, Calgary, AB, Canada

[2]Hotchkiss Brain Institute, Cumming School of Medicine, University of Calgary, AB, Canada

[3]McCaig Institute for Bone and Joint Health, Cumming School of Medicine, University of Calgary, AB, Canada

[4]Snyder Institute for Chronic Diseases, Cumming School of Medicine, University of Calgary, AB, Canada

[5]Department of Biochemistry and Molecular Biology, Cumming School of Medicine, University of Calgary, AB, Canada

[6]Department of Emergency Medicine, Warren Alpert Medical School & School of Engineering, Brown University, Providence, RI, USA

[7]Institute of Medical Education and Institute of Laboratory Medicine, University Hospital, LMU Munich, Germany

[8]Biomedical Engineering Department, University of Connecticut Health Center, Farmington, CT, USA

[9]Cell Biology and Anatomy, Cumming School of Medicine, University of Calgary, AB, Canada

[10]Physiology and Pharmacology, Cumming School of Medicine, University of Calgary, AB, Canada

*Corresponding Authors (Roman Krawetz: rkrawetz@ucalgary.ca and Antoine Dufour: antoine.dufour@ucalgary.ca)

Keywords: Tryptase, Proteoglycan-4, Lubricin, Osteoarthritis, Proteases, Lubrication, Proteomics, N-terminomics, Single-cell RNA-seq.

Figure 1

a

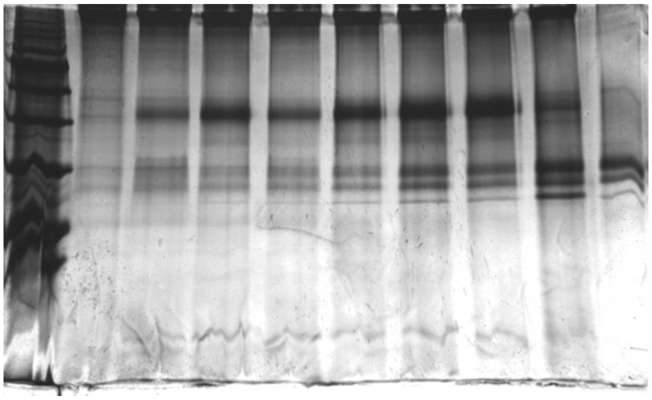

b

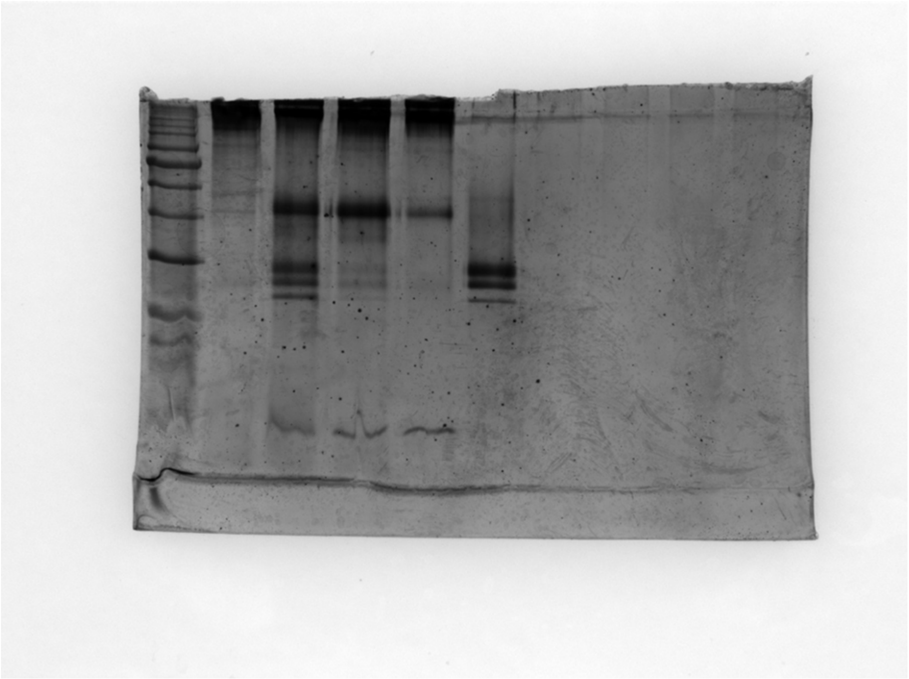

c

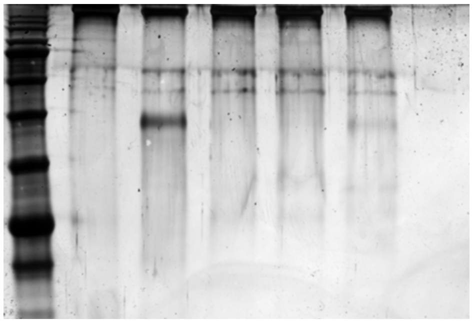

Supplement: Supplementary file 5 — Source Data [file 41467_2023_37598_MOESM5_ESM.zip › Source Data/source.pdf]
